# Reduction of chronic malnutrition for infants in Bogotá, Colombia

**DOI:** 10.1186/s12889-021-10620-3

**Published:** 2021-04-08

**Authors:** Paula Andrea Castro Prieto, Kenny Margarita Trujillo Ramírez, Sergio Moreno, Juan Sebastián Holguín, Diana María Pineda, Simón Tomasi, Andrea Ramirez Varela

**Affiliations:** 1 Population Health Axis, Fundación Santa Fe de Bogotá, Carrera 7b # 123-90, 110111, Bogotá, Colombia; 2 Centre d’Estudis Demogràfics, Universitat Autònoma de Barcelona, Barcelona, Spain; 3 Nutrición Social, Bogotá, Colombia; 4 Social Investment and Knowledge Generation, Fundación Éxito, Medellín, Colombia; 5 Faculty of Medicine, Universidad de los Andes, Bogotá, Colombia

## Abstract

**Background:**

According to the 2015 National Survey of the Nutritional Situation in Colombia the prevalence of stunting in children under 5 years of age was 10.8%. In terms of region, Bogotá, presented the highest prevalence rate (13%), a figure that exceeded national records. With the collaboration of local and national government, and nongovernmental it was decided to develop a pilot study involving a public health intervention with residents of Bogotá under 1 year of age with nutritional classification by anthropometry compatible with stunting risk or stunting.

**Methods:**

Pre-experimental, before and after study that sought to determine the magnitude of the change in nutritional status through a 10 months public health nutrition intervention in children under one-year-old residing in 3 prioritized territories of Bogotá.

**Results:**

The intervention comprised 1126 children living in the following territories in Bogotá: Kennedy, San Cristóbal, and Engativá. A total of 43.3% children presented delay in height for age, and 56.7% presented risk of short stature. In the final measurement, data were obtained from 686 children, identifying that 17% of the children progressed from stunting to a stunting risk and that 4.5% recovered their growth trajectory, achieving an adequate length for their age.

**Conclusion:**

That children classified as at risk or stunting at the beginning of the intervention showed an increased probability of approaching or being in the appropriate growth trajectory according to the length-for-age indicator after the intervention.

**Supplementary Information:**

The online version contains supplementary material available at 10.1186/s12889-021-10620-3.

## Background

Childhood chronic malnutrition corresponds to stunting, measured using the length-for-age indicator, and is classified as such when length is below 2 standard deviations (<− 2 SD) with respect to the median child growth from the World Health Organization (WHO). Worldwide, at the end of 2018, stunting affected 149 million children under five years of age and 4.8 million children in Latin America [1].

Delay in length is the consequence of poor nutrition, repeated infections, and inadequate psychosocial stimulation during the first 1000 days of life [2]. Length is a determinant of child development because it is not only a matter of centimeters gain but also of the development of all functions and organs in the body, including the brain, which has great repercussions on learning capacity [3].

Being stunted before 2 years of life and not overcoming it becomes a predictive factor of low school performance and the appropriation of skills at later ages, outcomes that negatively affect the economic development of both individuals and collectives in communities and countries [2]. In economic terms, stunting is related to productivity, with implications on the economic development of countries. It is estimated that children with stunting may lose two or 3 years of school and have 23% less income in their adult life, resulting in a 3% reduction in the gross domestic product (GDP) of countries at the national level [4].

Stunting, in addition to being the “best general indicator” of the level of well-being of children, is also a “reflection” of the social inequalities that affect a territory. It has been described that stunting “is a symptom of deficiencies of the past and an indicator of poverty in the future” [2].

According to UNICEF, in 2019, one in three children under 5 years of age in the world is not growing well because they suffer from stunting, acute malnutrition or overweight and, in some cases, manifest up to two of these forms of malnutrition. In addition, there are children who suffer from hidden hunger, that is, micronutrient deficiencies that seriously affect both their survival and growth and development in all stages of life [1].

Colombia is no exception, and according to the 2015 National Survey of the Nutritional Situation (Encuesta Nacional de la Situación Nutricional - ENSIN), the prevalence of stunting in children under 5 years of age was 10.8%, with a higher prevalence in boys compared to girls, 12.1% vs. 9.5%; In terms of region, Bogotá, presented the highest prevalence rate (13%), a figure that exceeded national records [5].

Given that Bogotá was the most affected region in the country and the negative consequences of stunting on children’s growth and development potential, an intervention to generate knowledge for action was needed. Due to, a public-private alliance was created between the local government (Mayor of Bogotá and its secretariats of health and social integration), a representative of the national government (Colombian Institute of Family Welfare, Instituto Colombiano de Bienestar Familiar - ICBF) and non-governmental organizations recognized in Colombia for their commitment to nutrition during childhood: Fundación Éxito and Fundación Santa Fe de Bogotá.

With the collaboration of these allies, it was decided to develop a pilot study involving a public health intervention with residents of Bogotá under 1 year of age with nutritional classification by anthropometry compatible with stunting risk or stunting. The general objective was to determine the effectiveness of an intersectoral public health intervention with the population under 1 year of age, classified as at risk of stunting and with stunting by anthropometry, residing in three prioritized territories of the Capital District.

The focus on chronic malnutrition is novel for research and intervention in Colombia, since the study and implementation of policies around malnutrition has focused primarily on acute malnutrition [6] which is of vital importance; however, to date no experiences focusing on chronic malnutrition have been reported.

The results of this study were used to develop a guide for the prevention, management and risks of chronic malnutrition as an event of interest in public health; the guide may serve as an instrument of public policy with scaling up potential in other territories.

This introduction is the first section of this paper, followed by presentation of the methods, data, results, contributions, and conclusions of the study.

## Methods

### Study design

This was a pre-experimental study, with before and after intervention analyses, to determine the magnitude of the change in nutritional status determined by anthropometry of children under 1 year of age residing in three prioritized territories of Bogotá, exposed to an intersectoral public health intervention for 10 months.

### Sample

To select the territories, the 2017 and 2018 databases from epidemiological surveillance systems were reviewed. Five of the 20 territories accounted for 47% of the cases and, Engativá, Kennedy and San Cristóbal, which are in different latitudes of the city, were identified as the three territories with the greatest number of chronic malnutrition cases [see Additional file 1].

The minimum ideal sample size was 650 children younger than 10 months of age with anthropometric nutritional classification compatible with a risk of short stature (Length /Age (HAZ) indicator cut-off point ≥ − 2 to < − 1) and/or chronic malnutrition (cut-off point < − 2) [see Additional file 2].

### Recruitment

The following inclusion criteria:

- Children with the length-for-age indicator (L/A) less than − 1 SD
- Children aged 10 months or younger at study entry
- Product of a full-term pregnancy (birth from 37 weeks or more)
- Resident of any of the three prioritized territories.

The defined exclusion criteria were as follows:

- Product of multiple pregnancies
- Place of residence different from Bogotá
- Any special health condition (disability)
- Congenital pathology
- Confirmed diagnoses of diseases requiring pharmacological treatment with hormone therapy and/or special diets preventing compliance with the recommendations of complete, balanced, sufficient, and adequate nutrition for healthy children
- Families not consenting study participation.

### Intervention model

**Fig. 1:**
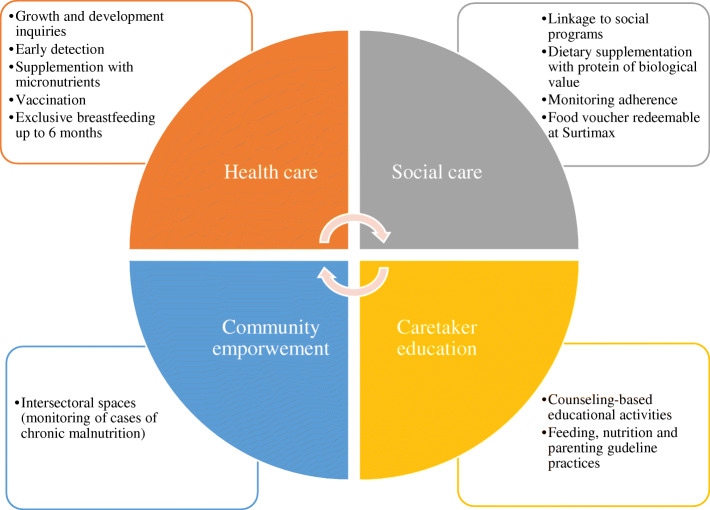
Intervention model of the study

The intervention was designed according to evidence-based recommended actions, adopted and regulated by Colombia [7] and, the Evidence-Based Clinical Practice Guide [8] to achieve adequate health and nutrition in early childhood. This intervention model (Fig. 1) focused on actions throughout the first 1000 days of life (from conception through the first 2 years of life), and included the following axes:

- *Health care:* provided specific information to families about the health care each child should receive according to their age and current condition. Individual needs were identified through baseline interviews.
- *Social care:* included actions to guide families to early education care and the social benefits available in each territory. As part of the development of this axis, a pedagogical food supplementation strategy was implemented through the delivery of redeemable vouchers in supermarkets that had to be exchanged in order to get a pre-established list of healthy foods (determined by nutritionists in the team). The foods obtained from the voucher should contribute 33% of the daily caloric requirements and 100% of the protein requirements, which would be additional to what the children received at home or in other social programmes. Compared to other sources of food supplementation, the voucher was used to provide nutritious foods that would strengthen the nutritional recovery of children, mainly associated with the contribution of protein of high biological value and energy from healthy sources [see Additional file 5].
- *Caregiver education*: considered as the linkage of all axes and actions. Therefore, face-to-face workshops were held for fathers, mothers and other caregivers regarding food and nutrition, parenting, and development guidelines, all specific to age groups and using innovative methodologies that would allow the greatest appropriation of knowledge to be applied for the care of children.
- *Community empowerment:* its purpose was to achieve the sustainability of the actions that were developed in the other components beyond the time of the study and the adherence to them to benefit more children in the territories. Its development included characterization and participation in the intersectoral workspaces of each territory undergoing the intervention and Bogotá as a city from the central government, in addition to the deployment of a communication strategy with various tools.

**Fig. 2:**
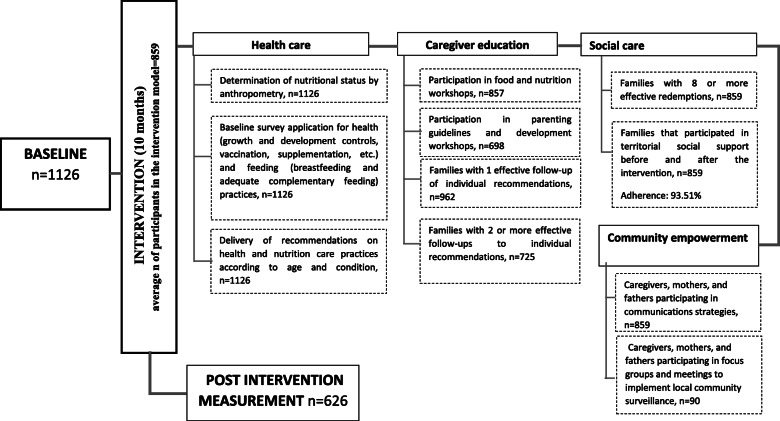
Distribution of study beneficiaries according to participation in the activities defined for the intervention model

The distribution of study beneficiaries according to participation in the activities defined for the intervention can be consulted in the Fig. 2.

### Variables

The *dependent variable* was the magnitude of the change in nutritional status determined by anthropometry in the length-for-age indicator (L/A). Appropriate length for age was defined as ≥ − 1 standard deviation (SD), risk of short stature was defined as ≥ − 2 and < − 1 SD, and delay in length was defined as < − 2 SD [9], as proposed by the WHO [10]. Nutritional status is defined as the dependent variable because the baseline status of the children was considered for the analysis, as it was different in all cases. Statistical power was present because it adjusts the follow-up measurement of each subject according to its baseline measurement [11].

The *independent variables* were determinants of the nutritional status of individuals:

- Feeding: through the intervention, parents and caregivers were educated to strengthen infant feeding practices. For children under 6 months the intervention focused on providing tools to promote the practice of exclusive breastfeeding and for children older than 6 months the focus was continuation of breastfeeding together with the adequate introduction of complementary feeding, as recommended by the WHO.
- Nutrition: weight and length.
- Health: history of pregnancy, tracking growth’p, vaccination, supplementation and home enrichment with micronutrients, educational messages.
- Social: redemption and use of food vouchers destined to strengthen the feeding of the breastfeeding mother and that children strengthen their complementary feeding; and participation/connection with social programmes; and
- Socioeconomic and demographic context: locality of residence, household income, female head of household, mother’s schooling, mother’s age and child’s age.

### Data collection

Collection of baseline information was conducted between May and July 2018. Post-intervention measurement was developed between May and August 2019.

Anthropometric measurements were taken with a weighing machine (Seca 876) and an infantometer (Seca 417), measurements were repeated three times (with each child) to ensure the precision and accuracy of the data. To collect information, a system engineer built a web application called azure (http://dntproyecto.azurewebsites.net/). The databases created for the study were anonymized for statistical analysis, reporting of results, and conclusions.

### Statistical analysis

A descriptive analysis was carried out to understand the sociodemographic characteristics of the sample, the consumption of food sources of protein, and determine the indicator of introduction of solid, semi-solid or soft foods [12].

To analyse the effects of the intervention on the nutritional status measured by anthropometry via L/A and the factors associated with L/A changes (comparing baseline and final post-intervention measurements), statistical analysis was performed using a *multivariate logistic regression model.*

For the purposes of the model, the dependent variable was constructed by comparing the nutritional status via the first and second L/A measurements (baseline and post-intervention measurements). A positive effect of the intervention was defined as presenting *length-for-age with a positive trend suggestive of an approach to the appropriate growth trajectory or presenting a L/A compatible with being in the normal growth trajectory*. The above required fulfilment of the following 2 conditions: 1) a comparison between the baseline and the final measurements and 2) having participated in all the components of the intervention (education, social assistance from the redemption of pedagogical food vouchers and health monitoring)*.*

The independent variables of the model were defined as follows (throughout the sample):

- Territory: Kennedy was used as an adjustment variable in the model as a reference category.
- Educational level of the mother: although the primary reference category presented a lower number of observations than the other categories.
- Socioeconomic variables: these were not included in the model because more than 95% of the sample had access to basic public services.
- Adherence to workshops: this variable was not included in the model because consolidated attendance at workshops by beneficiaries exceeded 95% [see Additional file 3].

Finally, a check of the multivariate logistic regression model assumptions was carried out to reaffirm that the assertions in the results were accurate. In doing so, some atypical points were removed from the model. Similarly, AIC: Akaike Information Criteria and BIC: Bayesian Information Criteria were developed. In addition, goodness-of-fit was assessed through a Hosmer and Lemeshow test and a linearity link test, showing that the model fits the data.

It should be clarified that it was assumed that the three territories presented different behaviours, therefore, the analysis was adjusted by territory membership, in order to reduce the variance explained by the membership of each territory.

Due to the high prevalence of the event of interest, the transformation of Odds ratio to prevalence ratios was used.

The statistical package used in the analysis was Stata 16 MP.

## Results

### Baseline

One thousand seven hundred fifty-two children were screened, of whom 1126 voluntarily agreed to participate in the study in the three prioritized localities. 42.81% of the sample lived in Engativá, while 30.02 and 27.18% lived in Kennedy and San Cristóbal, respectively (Table 1).

**Table 1:** demographic characteristic of the participants

Characteristic	Frequency	Percentage
Distribution of participants by location, baseline		
Engativá	482	42.81
Kennedy	338	30.02
San Cristóbal	306	27.18
Total	1126	100
Distribution of the participants by locality of residence, post-intervention final measurement		
Engativá	285	41.55
Kennedy	189	27.55
San Cristóbal	170	24.78
Other locations	42	6.12
Total	686	100
Distribution of participants according to nutritional status by anthropometry, length/age indicator, baseline		
Delay in length	487	43.25
Risk of short stature	639	56.75
Total	1126	100
Changes in the length-for-age indicator		
Chronic malnutrition (baseline)		
Chronic malnutrition	142	20.70
Risk of short stature	117	17.06
Adequate length	31	4.52
Risk of short stature (baseline)		
Chronic malnutrition	45	6.56
Risk of short stature	203	29.59
Adequate length	148	21.57
Total	686	100

Regarding gender, 52.84% of the children were male, and 47.16% were female. In terms of age group, 55.60% of children were younger than 6 months, and 44.40% were older than 6 months. The main caregiver was mothers (87.74%). A total of 40.59% of these mothers had completed their high school education.

For anthropometric nutritional status (Table 1), 43.25% of the children were classified with length delay for age, and 56.75% were at risk of short stature. Of the variables obtained to assess feeding practices, for exclusive breastfeeding, less than 4 out of 10 children (37.80%) younger than 6 months were exclusively breastfed; San Cristóbal (40.65%) was the territory with the highest prevalence of the practice, followed by Engativá (38.15%), and Kennedy (35.32%).

For adequate complementary diet, eight out of ten participants (81.0%) between 6 and 8 months had adequate introduction of food consistencies. The consumption of food sources of protein such as eggs was reported for 31.20% of the children, while meat, fish, chicken and other food sources of animal protein were consumed by 49.20% of the children.

### Post-intervention final measurement

For the final measurement performed at the end of the ten-month intervention, the legal caretakers of the participants were contacted. Data and post-intervention measurements were collected from 686 children (the reduction in the sample was due to transfers from the participants’ place of residence). In this phase, before and after comparisons were performed and for this analysis, children without final measurements were not included.

Data imputation was not conducted due to length being a biological variable that changes during childhood, even with stunting. Additionally, from the statistical point of view, a statistical power of 80% was achieved with 686 final participants. Therefore, the comparisons described below correspond to 686 children that have both baseline and final post-intervention measurements [see Additional file 2].

The geographic distribution of participants measured in the post-intervention period can be found on Table 1. Regarding gender, 47.23% were female, and 52.77% were male. The age distribution was as follows: 64.58% were children between 12 and 18 months, 22.16% were children older than 18 months, and 13.26% were children between nine and 11 months. Similarly, it was found that mothers were the main caregivers (71.87%). Of this group, almost a quarter (22.11%) reported having completed technical or technological studies.

In terms of nutritional status by anthropometry (Table 1) and [see Additional file 4], 17.06% of the children transitioned from stunting to risk of short stature, while 4.52% (31 children) advanced to an appropriate L/A. For the risk of short stature, 21.57% (146 children) progressed towards adequate L/A, 29.59% maintained a risk of short stature, and 6.56% reported stunting. For the weight-for-length indicator, 2.10% of children who reported a delay in length also presented a risk or excess weight, compared to 17.90% who presented this same condition at baseline.

For the variables related to feeding practices, continued breastfeeding was reported by 73.70% of the participants. In turn, at baseline, 29.20% reported consuming eggs, which are a source of protein of high biological value and are easy to access, and in the final post-intervention measurement, 83.32% reported consuming eggs. At baseline, 45.20% reported consuming other food sources of animal protein, while in the final measurement, this value increased to 90.21%. The redemption of the voucher achieved 93.51% adherence throughout the intervention.

In education terms, 80.9% beneficiary families had their comprehensive assessment of growth and development cards for their children and were able to explain their importance.

### Multivariate logistic regression model

#### Model results

**Fig. 3:**
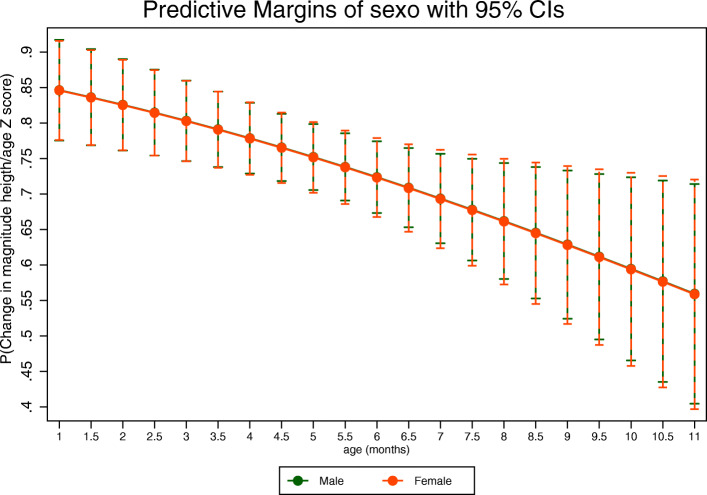
Marginals diagram stratified presenting the probability of approaching or being in the appropriate growth channel

According to Table 2 and Fig. 3, results suggest a decrease in the probability of approaching or being in the appropriate growth trajectory after the intervention (PR: 0.94 95% CI: 0.91–0.98) if children increased by 1 month in age.

**Table 2:** Multivariate logistic regression model presenting the probability of approaching or being in the appropriate growth

Variable	Coefficient (Prevalence ratio)	Confidence Interval (95%)	
Length-for-age z-score	0.81	0.70	0.92
**Age in months**	**0.94**	**0.91**	**0.98**
*Reference category (first child)*			
Second child or older	0.97	0.85	1.07
*Reference category (locality 1)*			
Locality 2	0.93	0.78	1.06
Locality 3	0.87	0.69	1.02
*Reference category (male)*			
Female	1.01	0.90	1.10
*Reference category (caregiver age < 40 years)*			
Older than or equal to 40 years	1.13	0.96	1.23
*Reference category (education level: elementary)*			
High school	1.03	0.80	1.18
Technical or technological or professional	1.06	0.82	1.20
Postgraduate	1.20	0.94	1.29
*Reference category (not exclusive breastfeeding)*			
Exclusive breastfeeding	0.99	0.74	1.16
*Reference category (non-continued breastfeeding)*			
Continued breastfeeding	1.01	0.76	1.17
*Reference category (no canned milk consumption)*			
**Consumption of canned milk**	**0.83**	**0.66**	**0.97**
*Reference category (no fruit consumption)*			
Fruits	0.93	0.59	1.16
*Reference category (no consumption of vegetables)*			
Vegetables	1.10	0.96	1.20
*Reference category (no consumption of protein sources)*			
Protein sources	1.09	0.94	1.19
*Reference category (no consumption of legumes)*			
**Legumes**	**1.13**	**1.05**	**1.20**
*Reference category (no minimum frequency of consumption)*			
Minimum frequency of consumption	1.12	0.96	1.21
*Reference category (no monitoring 1)*			
Monitoring 1	1.12	0.76	1.27
*Reference category (no monitoring 2)*			
Monitoring 2	0.76	0.16	1.23
*Reference category (less than 9 vouchers)*			
9 or more vouchers	0.92	0.76	1.04
*Reference category (≤ 1.0 CLMW)*			
**> 1.0 CLMW**	**1.09**	**1.00**	**1.16**
*Reference category (non-mother head of household)*			
**Mother head of household**	**1.09**	**1.00**	**1.17**
number of observations	571		

AIC null model = 779.679; BIC null model = 784.201; AIC full model = 622.322; BIC full model = 726.660; *n* = 571; Hosmer-Lemeshow test p value = 0.53; linktest *p* value = 0,028

In terms of the food component, children who were fed milk formula had a decreased probability of approaching or being in the proper growth trajectory after the intervention (PR: 0.83 95% CI: 0.66–0.97), compared to those who were not fed formula milk during the course of the intervention. Additionally, children who were fed legumes showed an increased probability of approaching or being in the proper growth trajectory after the intervention (PR: 1.13 95% CI: 1.05–1.20), compared to those who were not feed legumes during the course of the intervention.

Regarding sociodemographic data, the probability of approaching or being in the appropriate growth trajectory after the intervention increased (PR: 1.09 95% CI: 1.00–1.16) for children of households with an income greater than 1.0 current legal minimum wage (CLMW) compared to children of households with an income lower than 1.0 CLMW during the course of the intervention. This probability also increased (PR: 1.09 95% CI: 1.00–1.17) for children in families with female heads of households compared to children in families with male heads of households.

## Discussion

This study shows how a model of intersectoral intervention, to which a group of children under 1 year of age was exposed for 10 months, was able to change the nutritional status, as measured by anthropometry, i.e., L/A indicator, in 43.14% of the participants undergoing the intervention. Four children out ten moved positively in their growth trajectory; these results were based on final post-intervention measurements.

After the intervention, 4.52% of the children changed their nutritional status from chronic malnutrition to adequate length for age. These findings, when contrasted with the scientific evidence on health and nutrition interventions focused on reducing the delay in length in children under 5 years, are relevant because it has been described that those interventions of greater efficacy to reduce the prevalence of delay in length in children under 5 years are those that at least obtained a 3.0% change in the prevalence of length delay in the intervened population, with an exposure greater than or equal to 12 months [13] . In Bangladesh, CARE’s SHOUHARDO project, a nutrition intervention that links work with poverty and gender inequalities, achieved a 4.5 percentage point reduction in stunting in children aged 6–24 months [14].

In Amhara Ethiopia, an intervention (Child Caring Practices) was developed between 2004 and 2009 that included four components [1]: health [2], nutrition education [3], water [4], sanitation and hygiene, finding a 12.1% decrease in the prevalence of chronic malnutrition [15]. In Mexico, the “Oportunidades” conditional cash transfer program focused on providing fortified food, cash transfers, curative health services, and other benefits, it found children in intervention families aged less than 6 months grew 1.5 cm taller than children in comparison group families [16]. Also, a 10-year multisectoral intervention in sub-Saharan Africa, which included interventions in agriculture, health, education, and infrastructure, found that after 3 years the prevalence of chronic malnutrition in children under two was 43% lower than at the start of the program [17].

In respect to the magnitude of the change, the probability of length recovery was lower the older the age of the child. This result confirms, as several studies have described, the importance of implementing specific interventions on length delay during the most effective window of opportunity, that is, from gestation through the first 2 years of life [1, 18, 19].

The likelihood of approaching or entering an adequate growth trajectory after the intervention was found to increase when the child was in a female-headed household. A possible explanation for this result is provided by a study that argues that empowered mothers (through the female head of household, for example) have fewer time constraints to devote to their children, as well as having better mental health and more control over children and household resources, higher self-esteem, and better information and access to health services. This implies that empowered mothers take better care of themselves and their children, which is expected to have benefits for their children’s nutritional status [20].

Similarly, it has been shown that interventions that include timely education for caregivers for the age and current condition of the children, systematic monitoring, effective connection with health care and other sectors related to early childhood care, including basic sanitation and drinking water, developed in low- and middle-income countries are more effective for better outcomes related to child nutrition [13].

For example, at the end of the intervention, 80.9% beneficiary families had their comprehensive assessment of growth and development cards for their children and were able to explain their importance; their use demonstrates caregiver empowerment through exercising their rights and duties as citizens, benefiting them as a community. Necessary conditions for caregivers to effectively access health care relevant to the age of their children are key factors for the prevention and/or management of delayed length in the window of opportunity of early childhood [1].

It is necessary to mention that the educational strategy used for the intervention axis was counselling, whose principle is to work on the basis of the needs expressed by those who will be the subjects of the education using the skills that allow improving the communication process between the facilitators and the participants so that they acquire the necessary skills for informed decision making [21].

In this study, at the end of the intervention, seven out of ten children continued breastfeeding (73.7%) as part of their eating pattern; in comparison with the breastfeeding practice at baseline, improvement in practice was evident. Evidence has shown that using counselling contributes positively to practices related to the duration of exclusive and continued breastfeeding [22]. In agreement, a study identifying common breastfeeding problems in the postpartum period found that 98.3% of mothers considered breastfeeding education necessary [23].

Similarly, an improvement in the general practice of breastfeeding has been related as a function of maternal educational level and to mothers being immersed in protective environments and surrounded by community supporters [24]. These elements were also observed; most of the mothers had completed their high school education and a significant proportion, by the end of the intervention, had completed higher technical studies, a finding that suggests the importance of consolidating intersectoral strategies to favour the formal education of mothers and caregivers.

The probability of approaching or being in the appropriate growth trajectory, after the intervention, was reduced if the children were fed with formula milk compared to those who did not receive it. This result is consistent with other studies. A study conducted in public hospitals in Hong Kong found for a sample of 642 preterm children with low weight, those fed during their hospitalization with breast milk had a better z-score for length-for-age upon discharge than children fed formula milk because children fed formula have a higher risk of gastrointestinal infections that affect weight and length [25].

These results reaffirm breast milk providing nutrients children need for healthy growth and development during their first 2 years and beyond; therefore, it is necessary that social programs have as a priority the promotion and protection of this practice, as established by the WHO: exclusive breastfeeding during the first 6 months of life and adequate complementary feeding until 2 years or more [26].

According to the age of the children, 29.2% consumed eggs at baseline (older than 6 months), and 83.32% consumed eggs at the post-intervention measurement. That is, eight out of ten children were eating eggs as one of their main sources of protein. After the intervention, nine out of ten children (90.21%) had food sources of animal protein as part of their eating pattern. This result could be related to food voucher delivery, part of the social focus of the intervention model.

These vouchers were redeemed monthly by each beneficiary family in the study in a local supermarket. The redemption had a list of foods that included healthy food. This list was defined taking into account the recommendations for feeding for early childhood defined by the governing body of the sector for Colombia, ICBF [27]. Additionally, the proposed form of redemption favoured families having autonomy in decision-making for the purchase and preparation of food. This was mediated by the collaboration between the axes of education for caregivers and social care.

According to the evidence, the way to effectively intervene in length delays in early childhood requires comprehensive intersectoral work that encompasses cross-cutting actions that can account for most of the determinants of this condition, as the intervention developed in this study [19].

In relation to the sociodemographic results:

1. Family income plays a fundamental role in the recovery of stunting. A World Bank study argues that the link between income and nutritional status occurs mainly because households with higher income levels can invest more in consumption and variety of foods, in addition to having better quality of services and more resources to invest in the care of their children [28]. This relationship has been validated by different studies using different measures to determine income as monthly wages [28, 29] or assets in the home [30], among others.
2. The results obtained in the model in terms of the mother’s level of education were not consistent with the scientific evidence. Different authors have reported that children of more educated mothers have better results for the nutritional indicator length-for-age [2, 19, 31]. Education empowers women to make decisions that they would not be able to make in the absence of education, such as having fewer children or using health services more appropriately, leading to better physical and emotional development of their children [25, 32]. This relationship was not evident in the present study.

The intervention model implemented in the study is in line with several of the recommendations suggested by authors such as Butta et al., who refers to following effective actions in public health that make it possible to reduce length delays when implemented during early childhood: (i) folic acid supplementation in the preconception period; (ii) dietary supplementation to obtain a positive energy and protein balance in pregnant women; (iii) calcium supplementation for mothers; (iv) multiple micronutrient supplementation during pregnancy; (v) promotion of breastfeeding; (vi) adequate complementary feeding; (vii) administration of vitamin A; (viii) preventive zinc supplementation in children from six to 59 months; (ix) treatment of moderate acute malnutrition; and (x) treatment of severe acute malnutrition [33].

### Limitations

The sampling for this study was consecutive, and families were recruited mainly by mass communication strategies and the “snowball” technique. This sample determination did not allow us to extrapolate the results to the entire population of Bogotá. The intervention model developed and the results of the study directly pertain to the specific composition of the sample, mainly in terms of socioeconomic indicators; therefore, the magnitude of the change obtained in the Length/Age indicator for the beneficiaries of the study is specific to this group of children under the conditions that were treated.

## Conclusion

The risk of chronic malnutrition and/or chronic malnutrition in early childhood is a reversible condition if interventions are implemented in a timely manner and with intersectoral action, for which it is imperative to link the community itself as a key sector for direct action and to organise all actors and sectors that have missionary purposes with this population; this must be done to coordinate their multiple actions in the common approach to prevent and/or treat problems related to chronic malnutrition. Colombia, like other countries in the region, must continue its efforts to improve the visibility of this problem and make it a priority for the country’s development. Therefore, this research is a contribution to public health for the prevention and management of chronic malnutrition, as evidence in this area in Colombia is still scarce. Future research is invited to study the phenomenon over a longer period.

## Supplementary Information

**Additional file 1: Figure S4**. Number of chronic malnutrition or risk in children under 2 years of age in Bogotá.

**Additional file 2: Figure S5.** Sample size of intervention.

**Additional file 3: Figure S6.** Independent variables used for the construction of the multivariate logistic regression model.

**Additional file 4: Figure S7.** Change in magnitude length-age indicator.

**Additional file 5: Table S3.** Food offered on the voucher for each month.

## Data Availability

The datasets used and/or analyzed during the current study are available from the corresponding author on reasonable request.

## Abbreviations

GDP: Gross Domestic Product
UNICEF: United Nations Children’s Fund
ENSIN: National Survey of the Nutritional Situation (Encuesta Nacional de la Situación Nutricional)
ICBF: Colombian Institute of Family Welfare (Instituto Colombiano de Bienestar Familiar)
L/A: Length-for-Age indicator
WHO: World Health Organization
SD: Standard Deviation
AIC: Akaike Information Criteria
BIC: Bayesian Information Criteria
