# Correction to: Reduction of chronic malnutrition for infants in Bogotá, Colombia

**DOI:** 10.1186/s12889-021-12158-w

**Published:** 2021-11-15

**Authors:** Paula Andrea Castro Prieto, Kenny Margarita Trujillo Ramírez, Sergio Moreno, Juan Sebastián Holguín, Diana María Pineda, Simón Tomasi, Andrea Ramirez Varela

**Affiliations:** 1 Population Health Axis, Fundación Santa Fe de Bogotá, Carrera 7b # 123-90, 110111 Bogotá, Colombia; 2 Centre d’Estudis Demogràfics, Universitat Autònoma de Barcelona, Barcelona, Spain; 3 Social Investment and Knowledge Generation, Fundación Éxito, Medellín, Colombia; 4 Faculty of Medicine, Universidad de los Andes, Bogotá, Colombia

**Correction to: BMC Public Health 21, 690 (2021)**

**https://doi.org/10.1186/s12889-021-10620-3**

It was highlighted that in the original article [1] the affiliations of Kenny Margarita Trujillo Ramírez and Simón Tomasi were incorrectly captured. The original article has been updated.
