# Supplementary figures and images for: Reduction of chronic malnutrition for infants in Bogotá, Colombia
Source: BMC Public Health. 2021 Apr 8;21:690. doi: 10.1186/s12889-021-10620-3 (PMC8034142; doi:10.1186/s12889-021-10620-3)

**Figure 4. Number of chronic malnutrition or risk in children under 2 years of age in Bogotá**

**
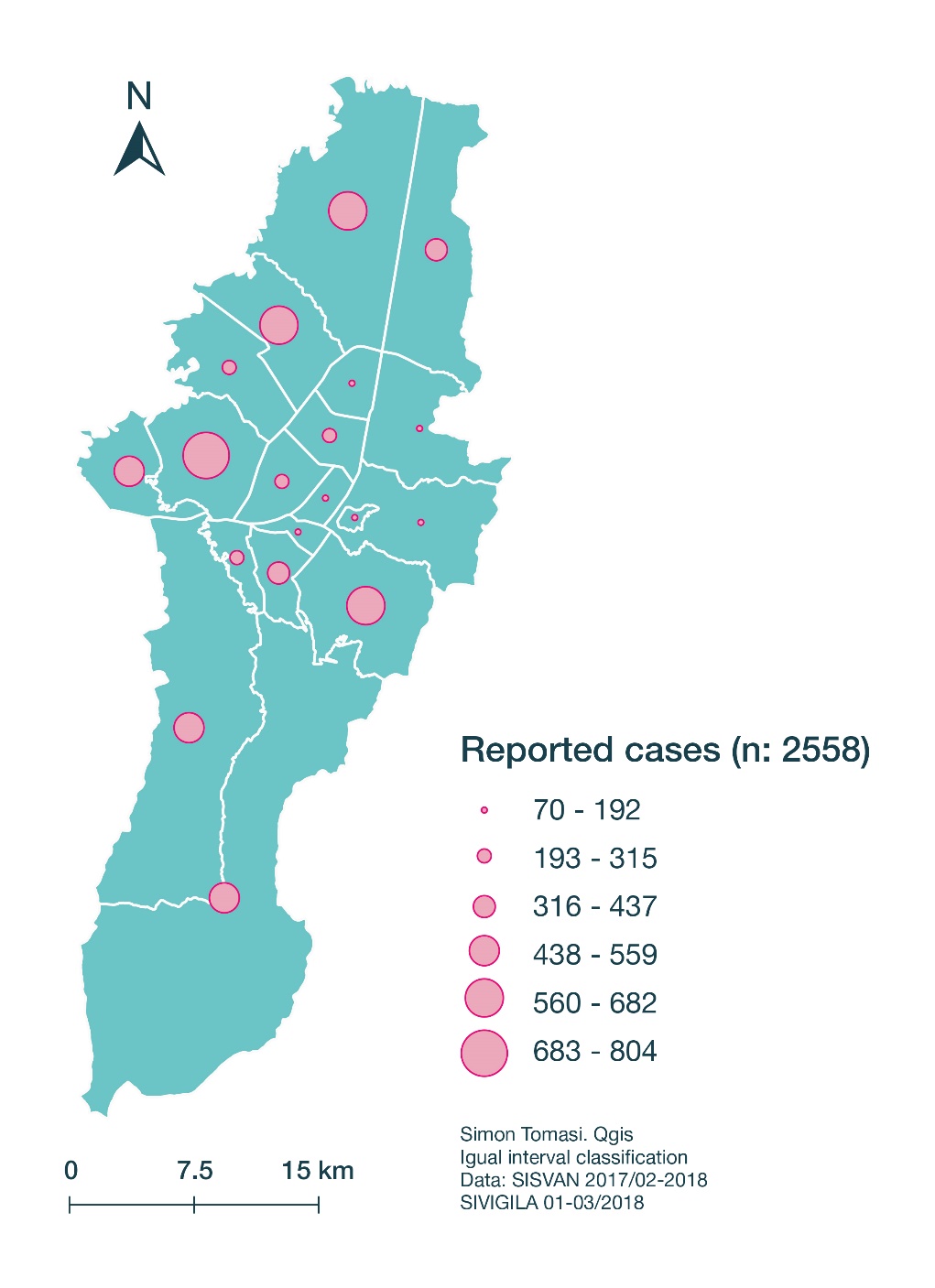
**

Supplement: Supplementary file 1 — Additional file 1: Figure S4. Number of chronic malnutrition or risk in children under 2 years of age in Bogotá. [file 12889_2021_10620_MOESM1_ESM.docx]

**Figure 2. Sample size of intervention**

**
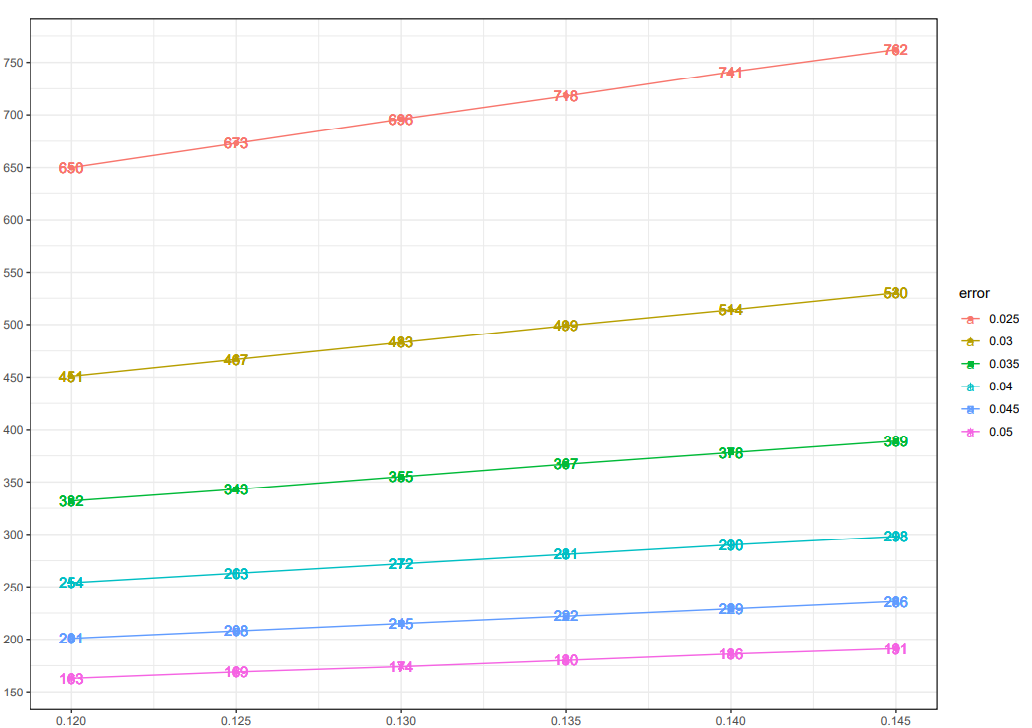
**

Estimated ratio

Sample size

Supplement: Supplementary file 2 — Additional file 2: Figure S5. Sample size of intervention. [file 12889_2021_10620_MOESM2_ESM.docx]

**Figure 7. Change in magnitude height-age indicator**

**
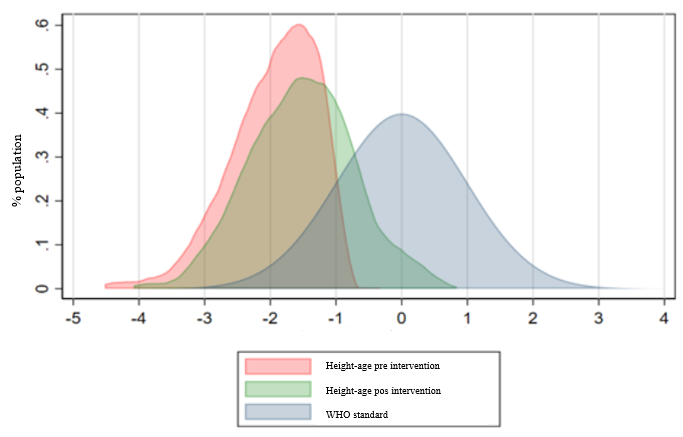
**

Supplement: Supplementary file 4 — Additional file 4: Figure S7. Change in magnitude length-age indicator. [file 12889_2021_10620_MOESM4_ESM.docx]
