# Supplementary material for: Reduction of chronic malnutrition for infants in Bogotá, Colombia
Source: BMC Public Health. 2021 Apr 8;21:690. doi: 10.1186/s12889-021-10620-3 (PMC8034142; doi:10.1186/s12889-021-10620-3)
Supplement: Supplementary file 3 — Additional file 3: Figure S6. Independent variables used for the construction of the multivariate logistic regression model. [file 12889_2021_10620_MOESM3_ESM.docx]

**Figure 6. Independent variables used for the construction of the multivariate logistic regression model**

Context and family

Social

Diet

Nutrition and health

- Number of pedagogical food bonds redeemed
- Locality of residence
- Household income
- Female head of household
- Mother's schooling
- Mother's age
- Child's age
- Child's birth order number
- Exclusive breastfeeding
- Continued breastfeeding
- Health monitoring
- Consumption of:
  - Formula milk
  - Protein source foods
  - Vegetables
  - Fruits
  - Legumes
  - Dairy derivatives
- Minimum frequency of consumption
