# Supplementary material for: Reduction of chronic malnutrition for infants in Bogotá, Colombia
Source: BMC Public Health. 2021 Apr 8;21:690. doi: 10.1186/s12889-021-10620-3 (PMC8034142; doi:10.1186/s12889-021-10620-3)
Supplement: Supplementary file 5 — Additional file 5: Table S3. Food offered on the voucher for each month. [file 12889_2021_10620_MOESM5_ESM.zip › Additional file 5R2.docx]

**Table 6. Food offered on the voucher for each month.**

| **Food** | **Quantity** |
| --- | --- |
| Eggs | 90 units |
| Beef | 2.5 pounds |
| Fruits | 8 pounds |
| Vegetables | 6 pounds |
| Red beans | 2 pounds |
| Cheese | 1 pound |
| Rice | 4 pounds |
| Sunflower seed oil | 1 liter |
